# The degradation of nucleotide triphosphates extracted under boiling ethanol conditions is prevented by the yeast cellular matrix

**DOI:** 10.1007/s11306-016-1140-4

**Published:** 2016-11-28

**Authors:** Andres Gil, David Siegel, Silke Bonsing-Vedelaar, Hjalmar Permentier, Dirk-Jan Reijngoud, Frank Dekker, Rainer Bischoff

**Affiliations:** 1 Department of Pharmacy, Analytical Biochemistry, University of Groningen, Antonius Deusinglaan 1, Building 3226, Room 601, 9713 AV Groningen, The Netherlands; 2 Biomolecular Sciences and Biotechnology Institute, Molecular Systems Biology, University of Groningen, Nijenborgh 4, 9747 AG Groningen, The Netherlands; 3 Department of Pharmacy, Interfaculty Mass Spectrometry Center, University of Groningen, Antonius Deusinglaan 1, 9713 AV Groningen, The Netherlands; 4 University Medical Center Groningen, Department of Pediatrics, Center for Liver, Digestive and Metabolic Diseases, University of Groningen, PO Box 30.001, 9700 RB Groningen, The Netherlands; 5 Department of Pharmacy, Pharmaceutical Gene Modulation, University of Groningen, Antonius Deusinglaan 1, 9713 AV Groningen, The Netherlands

**Keywords:** Metabolomics, Mass spectrometry, Nucleotides, Stability, Boiling ethanol extraction

## Abstract

**Introduction:**

Boiling ethanol extraction is a frequently used method for metabolomics studies of biological samples. However, the stability of several central carbon metabolites, including nucleotide triphosphates, and the influence of the cellular matrix on their degradation have not been addressed.

**Objectives:**

To study how a complex cellular matrix extracted from yeast (*Saccharomyces cerevisiae*) may affect the degradation profiles of nucleotide triphosphates extracted under boiling ethanol conditions.

**Methods:**

We present a double-labelling LC–MS approach with a ^13^C-labeled yeast cellular extract as complex surrogate matrix, and ^13^C^15^N-labeled nucleotides as internal standards, to study the effect of the yeast matrix on the degradation of nucleotide triphosphates.

**Results:**

While nucleotide triphosphates were degraded to the corresponding diphosphates in pure solutions, degradation was prevented in the presence of the yeast matrix under typical boiling ethanol extraction conditions.

**Conclusions:**

Extraction of biological samples under boiling ethanol extraction conditions that rapidly inactivate enzyme activity are suitable for labile central energy metabolites such as nucleotide triphosphates due to the stabilizing effect of the yeast matrix. The basis of this phenomenon requires further study.

**Graphical abstract:**

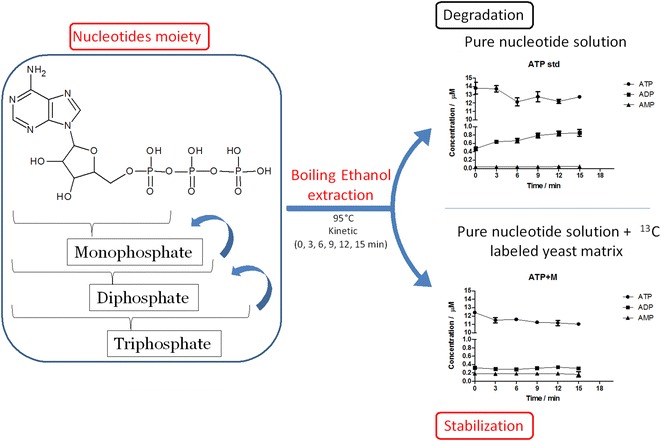

**Electronic supplementary material:**

The online version of this article (doi:10.1007/s11306-016-1140-4) contains supplementary material, which is available to authorized users.

## Introduction

Nucleotides are central carbon metabolites that are highly susceptible to enzymatic and/or non-enzymatic degradation. Exact knowledge of the concentrations of ATP, ADP and AMP is, for example, critical to calculate the energy charge of a cell which affects overall metabolism. Enzymatic turnover rates of cytosolic ATP and ADP are in the range of 1.5–2.0 mM s^−1^, and are affected by minimal changes in the cellular environment (Faijes et al. 2007; Villas-Bôas and Bruheim 2007; Vuckovic 2012). Consequently, instantaneous inactivation of enzymatic degradation processes (quenching) during sample preparation is essential to maintain nucleotide concentrations at the original levels (León et al. 2013; Lu et al. 2008; Vuckovic 2012). On the other hand matrix components, such as divalent cations, particularly Ni^2+^, Zn^2+^, Mn^2+^ and Cu^2+^ have been shown to catalyze the non-enzymatic hydrolytic degradation of ATP, GTP, ITP, and CTP (Gil et al. 2015). When these nucleotides are incubated in the presence of Cu^2+^ at 50 °C and pH 5.0–8.0, there is a decrease in half-lives from approximately 10 days to 46.2 min for ATP, 385 min for GTP, 770 min for ITP, and 2310 min for CTP (Amsler and Sigel 1976; Sigel and Amsler 1976; Sigel and Hofstetter 1983). Thus, besides enzymatic transformation during sample preparation, the hydrolysis of nucleotide triphosphates caused by components of the sample matrix deserves attention, since it may affect the final quantitative results of metabolomics studies.

Several studies dealing with nucleotide stability during sample preparation for metabolomics, have focused on the development of new types of quenching/extraction processes and the evaluation of residual enzymatic activity (Canelas et al. 2008, 2009; Loret et al. 2007; Vuckovic 2012). Despite these efforts, there is a lack of knowledge regarding the changes induced by the physicochemical conditions to which samples are exposed at the processing stage. Usually, at the sample processing stage, working temperatures are in the range of 4–25 °C for sampling, and −40 to 95 °C for quenching/extraction, while the pH can vary between 2 and 8. Furthermore, sample preparation may take up to 24 h in chemical environments rich in water and oxygen, as well as in the presence of light (Gil et al. 2015; Siegel et al. 2014). All of these conditions may induce purely chemical degradation processes that might be exacerbated by components of the sample matrix.

Boiling ethanol, first introduced as an approach to extract metabolites from yeast, has become one of the most frequently used methods for metabolomics studies in other biological systems (Airoldi et al. 2015; Dunn and Winder 2011; Gonzalez et al. 1997). While several researchers have used this type of extraction with different modifications, according to particular needs (Buescher et al. 2010; Canelas et al. 2009; Faijes et al. 2007; Paglia et al. 2012; Sellick et al. 2010), there is little information regarding the stability of critical molecules, including nucleotides, under such harsh extraction conditions. This issue has been addressed by evaluating the stability of the four major nucleotide triphosphates present in cells (ATP, GTP, UTP, and CTP) showing that there is significant degradation of these metabolites, caused by the extraction procedure (Gil et al. 2015).

The purpose of this study was to determine how a complex cellular matrix extracted from yeast (*Saccharomyces cerevisiae*) may affect the degradation profiles of nucleotides under boiling ethanol conditions. To this end we used a double-labelling approach with ^13^C-labeled yeast extract as complex surrogate matrix and ^13^C^15^N-labeled nucleotides as internal standards. To get a better understanding of the effect of the yeast matrix on nucleotide triphosphate degradation, we evaluated the effect of a range of matrix components on stability.

## Materials and methods

For chemicals, the calibration with ^13^C^15^N-labeled nucleotides, and the statistical analysis see Electronic Supplementary Material.

### Generation of ^13^C-labeled yeast cellular matrix

Preparation of ^13^C-labeled yeast cell extracts was done according to Buescher et al. (2010), with slight modifications. *S. cerevisiae*, strain YSBN6 (MATa; genotype: FY3 ho::HphMX4 derived from the S288C parental strain) was grown on glucose minimal medium (Verduyn medium) containing (per L): 20 g ^13^C-glucose, 5 g (NH_4_)_2_SO_4_, 3 g KH_2_PO_4_, 0.5 g MgSO_4_·7H_2_O, 4.5 mg ZnSO_4_·7H_2_O, 4.5 mg CaCl_2_·2H_2_O, 3 mg FeSO_4_·7H_2_O, 2.5 mg inositol, 1.5 mg EDTA, 1 mg H_3_BO_3_, 1 mg MnCl_2_·4H_2_O, 0.4 mg NaMoO_4_·2H_2_O, 0.3 mg CoCl_2_·6H_2_O, 0.3 mg CuSO_4_·5H_2_O, 100 µg KI, 100 µg thiamine, 100 µg Ca-pantothenic acid, 100 µg nicotinic acid, 100 µg pyridoxine, 20 µg p-aminobenzoic acid, and 5 µg biotin, buffered with 10 mM potassium phthalate to pH 5.0 (Verduyn et al. 1992). Cultures of 12 × 85 mL were incubated in 1 L flasks at 30 °C with shaking at 250 rpm and grown to OD_600_ = 3.5 ± 0.2. 0.213 g of ^13^C-labelled glucose was added 10 min prior to harvesting. For harvesting, 80 mL of culture were mixed with 320 mL of methanol, precooled to −80 °C. The methanol—culture mix was kept below −40 °C in a cold ethanol/ethylene glycol bath (1:1 mix) cooled with dry ice. Cells were separated by centrifugation at −9 °C and 2500 g for 15 min and the pellet was kept cold in dry ice prior to extraction.

Extraction was done as previously described (Buescher et al. 2010) with slight modifications. Briefly, samples were extracted twice with 350 mL of 60% (aq) ethanol for 2 min at 78 °C. After each extraction step, the mixture was centrifuged for 2 min at 13,000×*g* and 4 °C by means of a JLA-10.500 fixed angle rotor in a Beckman Coulter J2 centrifuge (Beckman Coulter, Inc., USA) and the supernatant was collected. The pooled supernatants were frozen in liquid nitrogen and the solvent removed in a VaCo2 freeze dryer (Zirbus Technology, Bad Grund, Germany). The residue was resuspended in extraction solvent (ethanol 60% aq) and the resulting solution was aliquoted and stored at −80 °C until further use.

### In-house production of ^13^C^15^N-labeled nucleotide di- and monophosphates

**Fig. 1:**
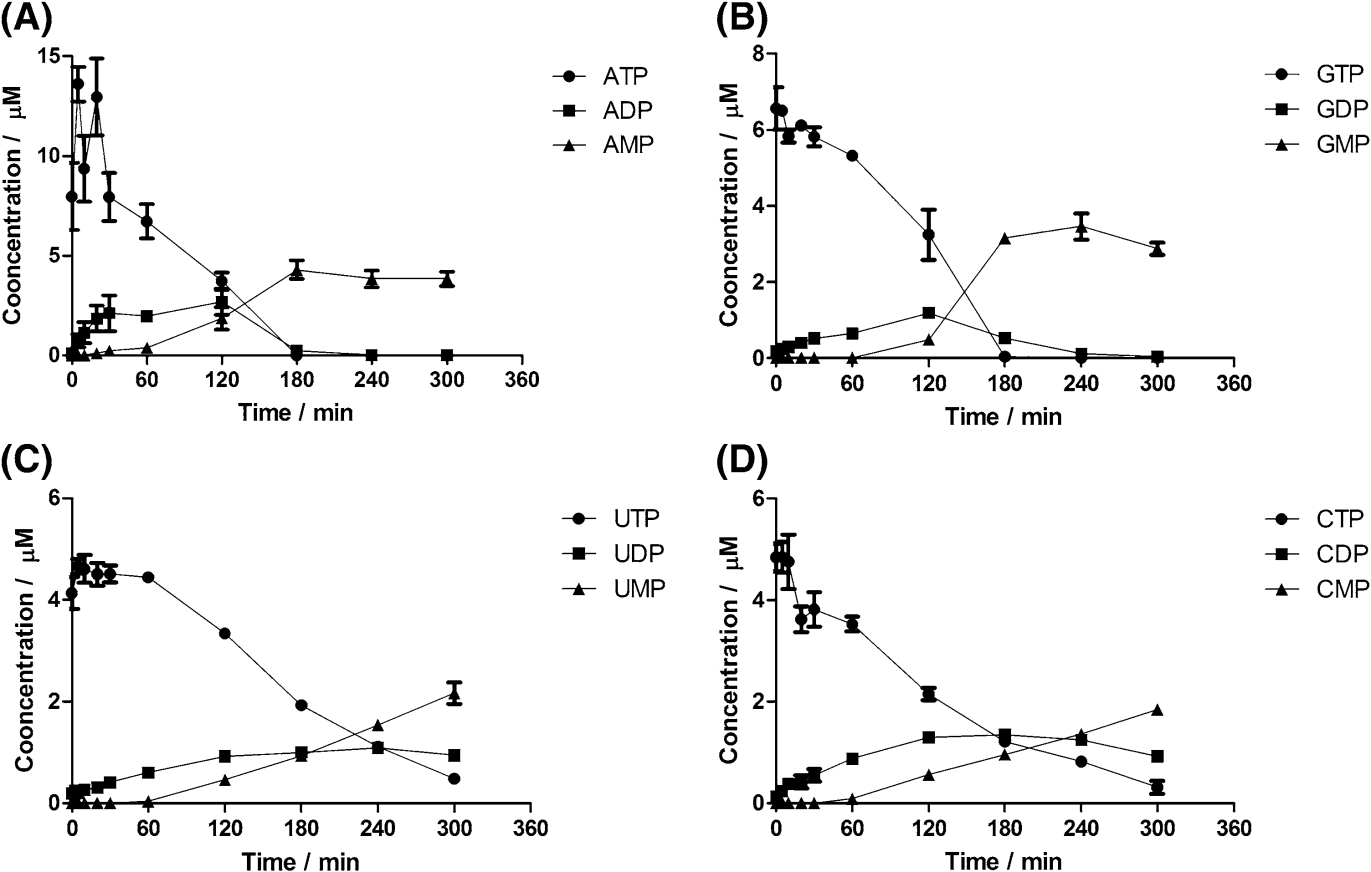
Kinetic profile of nucleotide triphosphate degradation in 75% (aq.) boiling ethanol. **a** ATP, **b** GTP, **c** UTP and **d** CTP. Each time point represents the mean of four independently prepared samples (±SD)

Labelled nucleotide di- and monophosphate internal standards were produced by a controlled degradation of ^13^C^15^N-labeled ATP, GTP, CTP and UTP. Briefly, 800 µL of 75% (aq) ethanol preheated at 95 °C were added to 200 µL of ^13^C^15^N-labeled nucleotide standard solutions at 100 mM. The mixture was transferred to 1.5 mL vials and incubated at 95 °C for 130, 200 and 220 min for ATP/GTP, CTP and UTP, respectively, since at these time points similar concentrations of the original compounds and the main degradation products (i.e. the di- and monophosphates) were obtained (Fig. 1). Reactions were stopped by snap-freezing in liquid nitrogen. The vials were thawed and excess solvent evaporated under a stream of nitrogen at room temperature. The residues were resuspended in 1 mL of ultrapure water and mixed to form a master solution of ^13^C^15^N-labeled nucleotides (tri-, di- and monophosphates) that was used as an internal standard for quantitative purposes in the remaining experimental work. Extracted-ion chromatograms (EIC) for the ^13^C^15^N-labeled tri-, di-, and monophosphates were compared with commercially available unlabeled compounds by detecting their respective exact mass-to-charge ratios (*m/z*) in high-resolution mode with an extraction window of 5 ppm. The internal standards showed identical MS/MS spectra (except for the mass shift) and retention times as the unlabeled nucleotides (ATP, ADP, AMP, GTP, GDP, GMP, CTP, CDP, CMP, UTP, UDP and UMP) (Fig. S1).

### LC–MS

Nucleotides were separated in the HILIC mode using a Luna NH_2_ column (3 µm, 100 × 2 mm; Phenomenex) on an Acquity UPLC system (Waters, Manchester, UK). Mobile phases consisted of a mixture of 5 mM ammonium acetate in water at pH 9.9 (eluent A) and acetonitrile (eluent B). Linear gradient elution went from 30% eluent A to 99% eluent A in 8 min, followed by isocratic elution at 99% eluent A until 14 min. A conditioning cycle of 6 min with the initial proportions of eluents A and B was performed prior to the next analysis. The column temperature was set at 20 °C, the flow rate was 0.25 mL/min, and the injection volume per sample was 10 µL.

Mass spectrometry detection was performed using a Waters Synapt G2-Si high-resolution Q-TOF mass spectrometer. The analytes (nucleotide triphosphates) and their respective degradation products (nucleotide di- and monophosphates) were detected by electrospray ionization in negative mode (ESI^−^). Nitrogen and argon were used as desolvation and collision gas, respectively. Data were acquired over the *m/z* range of 50–1200 Da, with the source temperature set at 150 °C, desolvation temperature at 400 °C, cone voltage at 30 V and capillary voltage at 2000 V. MS^E^ data acquisition was used in centroid and sensitivity modes. The collision energy potential setting was 2 V for the low-collision energy scan, and 10–30 V for the high-collision energy scan. As a result, for each sample, both precursor ion and fragment ion information was obtained from a single LC injection. The system was equipped with an integral LockSpray unit with its own reference sprayer that was controlled automatically by the acquisition software to collect a reference scan every 10 s lasting 0.3 s. The LockSpray internal reference used for these experiments was a 0.2 ng/µL leucine-enkephalin solution (*m/z* 554.2615 in negative ion electrospray mode) infused at 10 µL/min. Separation (UPLC) and detection (MS) systems were controlled by MassLynx 4.1 software (Waters, Manchester, UK).

### Degradation/interconversion of nucleotide triphosphates

The kinetic profiles of the degradation/interconversion for pure solutions of ATP, GTP, CTP and UTP were assessed under boiling ethanol extraction conditions as used for sample preparation in metabolomics studies. Briefly, tubes containing 496 µL of 75% (aq) ethanol were preheated at 95 °C for 5 min, followed by the addition of 4 µL of each nucleotide standard solution (500 µM) and vigorous mixing. To follow the kinetics of the degradation reactions the mixture was incubated at 95 °C under shaking for 0, 3, 6, 9, 12, and 15 min. Reactions were stopped by snap-freezing in liquid nitrogen and samples were stored on dry ice. Subsequently, samples were thawed and 20 µL of the ^13^C^15^N-labeled internal standard solution were added for quantitative analysis. Excess solvent was evaporated under a stream of nitrogen without heating. Finally, samples were reconstituted in 200 µL of acetonitrile–water 70:30 and stored at –40 °C until analysis by LC–MS. The influence of a yeast cellular matrix on the degradation/interconversion of nucleotides was assessed by adding 70 µL of the ^13^C-labeled matrix to the reaction mixture and continuing the experimental procedure as described above.

### Evaluation of selected matrix components on the degradation/interconversion of nucleotide triphosphates

To study the impact of components usually found in the yeast matrix, the effect of the whole culture medium (carbon source, vitamins, macro and micro elements, etc.) and the influence of particular groups of compounds/metabolites on the degradation/interconversion of nucleotide triphosphates during boiling ethanol extraction was assessed (Table 1). Briefly, the experimental procedure described in Sect. 2.4 was repeated, but instead of the ^13^C-labeled yeast cellular matrix, 70 µL of Verduyn culture medium or 4 µL of solutions “A” to “H” (Table 1) were added to the reaction mixture. After the experimental procedure the final concentration of all components was 10 µM in a volume of 200 µL. Compounds to be tested were divided into eight groups containing approximately ten compounds each, mainly comprising major central carbon metabolites including amino acids, organic acids, sugar phosphates, coenzymes, etc. (Table 1). Compounds with identical masses were assigned to different groups, so that no group contained two metabolites with masses closer than 2 Da, thus avoiding overlapping isotopic peaks.

**Table 1:** Standard solutions to simulate central carbon metabolome and some yeast xenobiotic compounds

Sol A	Sol B	Sol C	Sol D	Sol E	Sol F	Sol G	Sol H
2,3-biphosphoglycerate	2-phosphoglycerate	6-P-Gluconate	ATP	4-nitrophenol	Galactose-1-phosphate	2,2-dimethylsuccinate	Glucose
2-ketoglutarate	Glucose-1-phosphate	ADP	cAMP	Benzaldehyde	Niacin, Vitamin B_3_	Glycerate	AMP
*cis*-Aconitate	Glyceraldehyde-3-phosphate	Mannose-6-phosphate	d-Xylulose	Benzyl alcohol	Coumarin	Isocitrate	Citrate
3-phosphoglycerate	Lactate	Acetyl-CoA	Fructose-1-phosphate	Caffeine	Malonyl-CoA	Fumarate	Aspartate
Ribose-5-phosphate	Ribulose-5-phosphate	Fructose 1,6-Bis-phosphate	GDP	Choline	Riboflavin, Vitamin B_6_	Itaconate	Glutamate
Fructose-6-phosphate	Oxaloacetate	GTP	Glucosamine-6-phosphate	Glucose-6-phosphate	Phosphocholine	Arginine	Glutamine
Dihydroxyacetone-phosphate	NAD^+^	PEP	Phenylalanine	Salicylate	Acetoacetyl-CoA	Malate	Diaminopimelate
Ketoisoleucine	Shikimate	Biotin	Sedoheptulose-7-phosphate	Urate	Coenzyme B_12_	Methylmalonate	Oxalate
Piruvate	CoA	NADH	Succinyl-CoA	Spermine		Fructose	Succinate
NADPH	FAD	d-Ribose	NADP^+^	Ascorbate		GABA	Glyoxylate

## Results and discussion

### Production of ^13^C^15^N-labeled nucleotide di- and monophosphates

Quantitative mass spectrometry in highly complex biological matrices necessitates the use of stable-isotope-labeled internal standards, since ion suppression effects may affect quantification (Wu et al. 2005). While isotopically labeled nucleotide triphosphates (^13^C^15^N-labeled ATP, GTP, CTP and UTP) are commercially available, the respective ^13^C^15^N-labeled di- and monophosphates have to be prepared. To generate the missing standards, we subjected nucleotide triphosphates to controlled degradation at 95 °C for 130, 200 and 220 min for ATP/GTP, CTP and UTP, respectively, to reach comparable concentrations for each nucleotide (Fig. 1). Comparison of EICs with commercially available unlabeled compounds by LC–MS in high-resolution mode confirmed their identity (Fig. S1).

### LC–MS method performance and validation

Chromatographic conditions were optimized on the basis of peak resolution, baseline drift, and retention time resulting in separation of the 12 nucleotide phosphates into 3 groups in 10 min based on the number of phosphate groups in the hydrophilic interaction liquid chromatography (HILIC) mode at pH 9.9 (Fig. S1). The first group of eluting nucleotides consisted of the monophosphates followed by the di- and triphosphates, respectively. Similar separation patterns have been reported using ion-pairing chromatography albeit with longer retention times (Coulier et al. 2006) or less efficient separation (Seifar et al. 2009).

Quantitative isotope-dilution LC–MS analyses were performed with ^13^C^15^N-labeled nucleotide tri-, di- and monophosphates as internal standards. Calibration curves were obtained by plotting peak area ratios (unlabeled/^13^C^15^N-labeled nucleotides) against concentrations of the added unlabeled nucleotides (Figs. S2, S3). Method validation was performed by evaluating linearity (Table S1; r^2^ ≥ 0.9962), intra-day variability (repeatability), inter-day variability (intermediate precision) and accuracy. The limit of quantification (LOQ) was set to 9.8 nM, the lowest point on the calibration curve where the analyte responses were at least 5-times higher than the blank and where the coefficient of variation (CV) was less than 20% in accordance with international guidelines (EMA 2012; HSS–FDA 2013). Repeatability was assessed by testing 5 independently prepared samples that were spiked with each nucleotide at 0.625 µM and measured 8 times in one batch. Similar samples were prepared and measured on three different days to assess intermediate precision and accuracy. For all the nucleotides measured in this study, CV was lower than ±15% and accuracy higher than 95%, satisfying validation criteria for repeatability, intermediate precision and accuracy (Table S1). The validation results established the suitability of the method for the evaluation of nucleotide degradation under different conditions.

### Effect of the extraction procedure on the stability of nucleotides

**Fig. 2:**
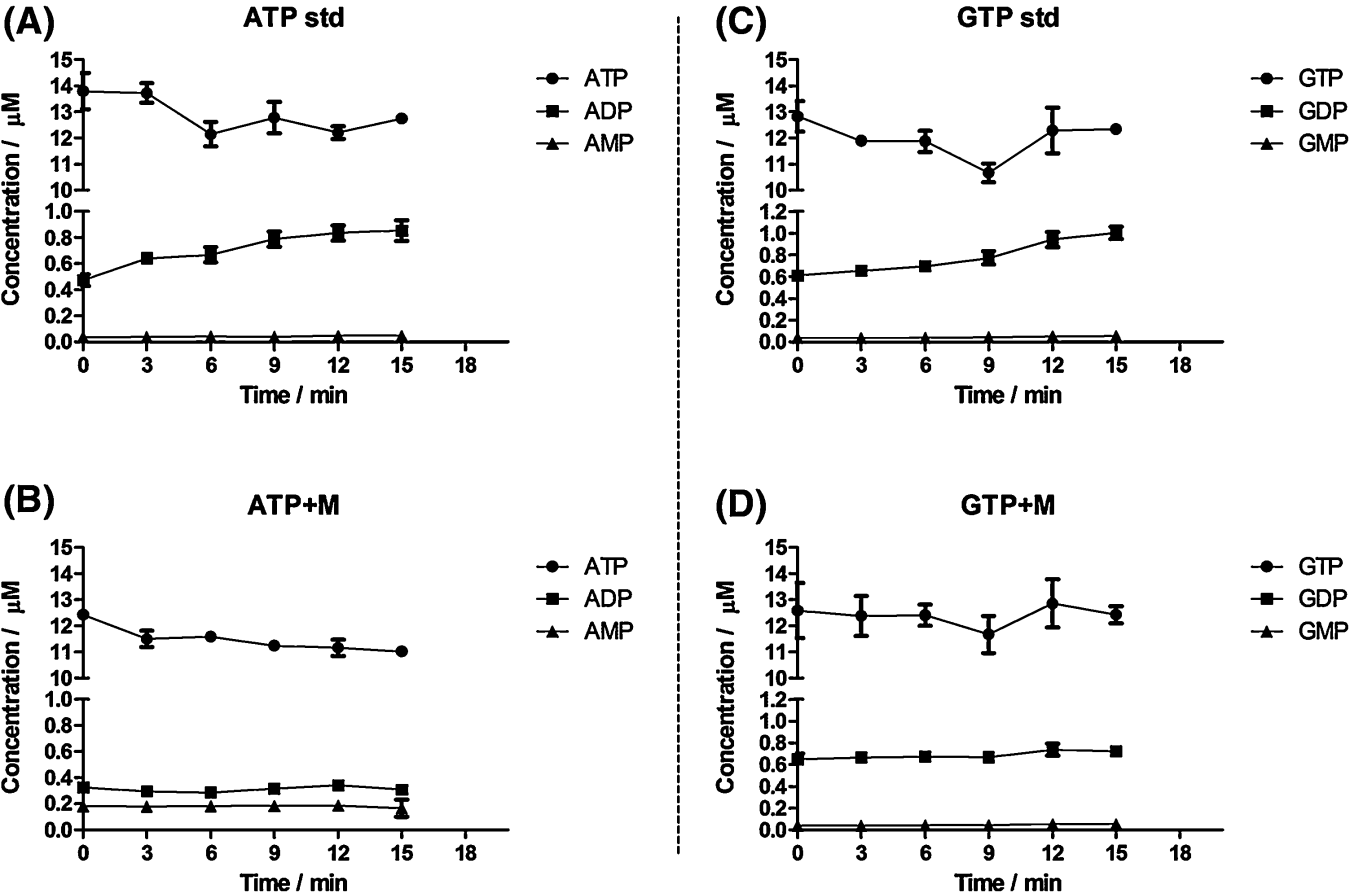
The yeast cellular matrix prevents degradation of **purine** nucleotide triphosphates during extraction in 75% (aq.) boiling ethanol. **a** ATP and **c** GTP extractions in the absence of the yeast cellular matrix. **b** ATP and **d** GTP extractions in the presence of the ^13^C-labeled yeast cellular matrix. Each time point represents the mean of four independently prepared samples (±SD)

**Fig. 3:**
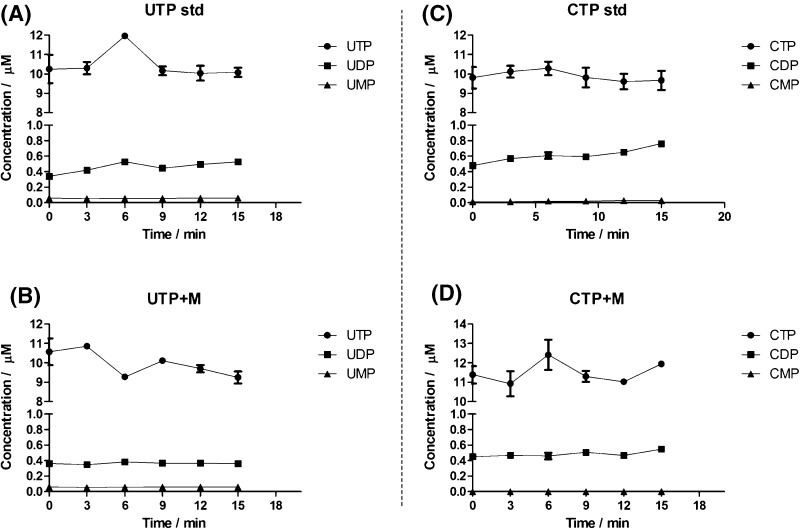
The yeast cellular matrix prevents degradation of **pyrimidine** nucleotide triphosphates during extraction in 75% (aq.) boiling ethanol. **a** UTP and **c** CTP extractions in the absence of the yeast cellular matrix. **b** UTP and **d** CTP extractions in the presence of the ^13^C-labeled yeast cellular matrix. Each time point represents the mean of four independently prepared samples (±SD)

Whereas enzymatic conversion of metabolites has been studied in great detail, nonenzymatic, chemical conversions have received comparatively little attention. By assessing the stability of nucleotide triphosphates under the conditions of a boiling ethanol extraction, one of the most frequently used methodologies for metabolomics studies, we specifically asked the question whether degradation may be affected by other components in the sample matrix. Extracting pure solutions of the nucleotide triphosphates with ethanol at 95 °C resulted in the degradation of purine and pyrimidine nucleotide triphosphates with a steady, time-dependent increase in the concentration of ADP and GDP (Fig. 2a, c) and of UDP and CDP (Fig. 3a, c). ADP was increased by 80.6% (0.473 to 0.854 µM), GDP by 63.4% (0.614 to 1.003 µM), UDP by 54.6% (0.341 to 0.526 µM) and CDP by 64.9% (0.477 to 0.762 µM) after 15 min incubation (*p* < 0.05; ANOVA). While there was a trend towards decreasing concentrations for the nucleotide triphosphates (Figs. 2, 3), these changes were not significant (*p* > 0.05; ANOVA). Therefore, changes in the concentration of nucleotide diphosphates are more sensitive indicators of nucleotide triphosphate hydrolysis. Although the concentration of nucleotide monophosphates (AMP, GMP, CMP and UMP) showed a tendency to increase as well, these increases were not statistically significant (*p* > 0.05; ANOVA) after 15 min (Figs. 2a, c, 3a, c). However, a significant increase in the concentration of the monophosphates was seen after 60 min at 95 °C in boiling ethanol (Fig. 1). According to the exhaustive degradation profiles shown in Fig. 1, in order to see a clear and statistically significant (*p* < 0.05) decrease in the concentration of nucleotide triphosphates and a corresponding increase in the concentration of nucleotide di- and monophosphates, the duration of the experiment at 95 °C should be extended well beyond 15 min. Even though time and temperature are the most common variables when conducting an extraction of samples with boiling ethanol for metabolomics studies, the temperature range generally applied with this extraction approach lies between 78 and 95 °C, and the time of extraction usually ranges from 1 to 15 min (Faijes et al. 2007; Canelas et al. 2009; Buescher et al. 2010; Sellick et al. 2010; Paglia et al. 2012 and Gil et al. 2015). Therefore, the lack of correlation between the decrease in concentration of nucleotide triphosphates and the increase in nucleotide di- and monophosphates may be due to the limited time of thermal exposure. Our results are consistent with a report by Daniel et al. (2004), who established the degradation kinetics for ATP, ADP and AMP and found similar kinetic profiles for degradation under aqueous conditions for 180 min at 95 °C. These authors also measured ATP stability in 90% (aq.) glycerol, an environment more similar to the 75% (aq.) ethanol that we used, and found them to be similar. It is important to note that an increase in the concentration of one metabolite due to the degradation of another metabolite (interconversion) is not corrected for by stable isotope dilution mass spectrometry, which can only compensate for decreases. This is a limitation of the stable isotope dilution approach in metabolomics.

### Effect of a complex cellular matrix on the stability of nucleotides

Since pure nucleotide triphosphates were degraded when extracted with boiling ethanol, we studied how a complex cellular matrix extracted from yeast (*S. cerevisiae*) may affect degradation kinetics using a uniformly ^13^C-labeled yeast extract as surrogate matrix and ^13^C^15^N-labeled nucleotides as internal standards. Incubation of unlabeled ATP, GTP, UTP and CTP with the ^13^C-labeled yeast matrix at 95 °C for 15 min showed a significant decrease in the degradation kinetics indicating a stabilizing effect of the matrix (Figs. 2b, d, 3b, d). In fact, the concentration of ADP, GDP, UDP and CDP as well as that of AMP, GMP, UMP and CMP remained constant during the 15 min incubation (*p* > 0.05; ANOVA). Next to the stabilizing effect of the yeast matrix, we found that the concentration of ADP was decreased at t = 0 min (0.473 to 0.325 µM; −68.6%) and that of AMP was increased (0.032 to 0.182 µM; +568.7%) in comparison to the initial concentration in the absence of the yeast matrix (Fig. 2a, b). While the increase in AMP may be explained by the conversion of other metabolites in the yeast matrix generating AMP (see next section), it is currently unclear how to explain the decrease in ADP concentration.

Since metabolite extractions with boiling ethanol are generally performed without the addition of buffers (Canelas et al. 2009; Faijes et al. 2007; Paglia et al. 2012; Sellick et al. 2010), we did not buffer the pure nucleotide solutions for our studies. However, trying to explain our results, we measured the pH of samples with and without the addition of the ^13^C-labeled yeast extract but found no difference (pH range = 5.8–6.0), indicating that stabilization of nucleotide triphosphates was not due to a pH effect. Earlier Gonzalez et al. (1997) suggested that differential metabolite extraction yields, particularly for ATP and related compounds, may be related to either compartmentalization and/or tight association with macromolecules rather than to their inherent stability in the extracting agents. It is unlikely that such effects play a role during the extraction with boiling ethanol.

### Influence of the culture medium on the stabilization/degradation of nucleotides

**Fig. 4:**
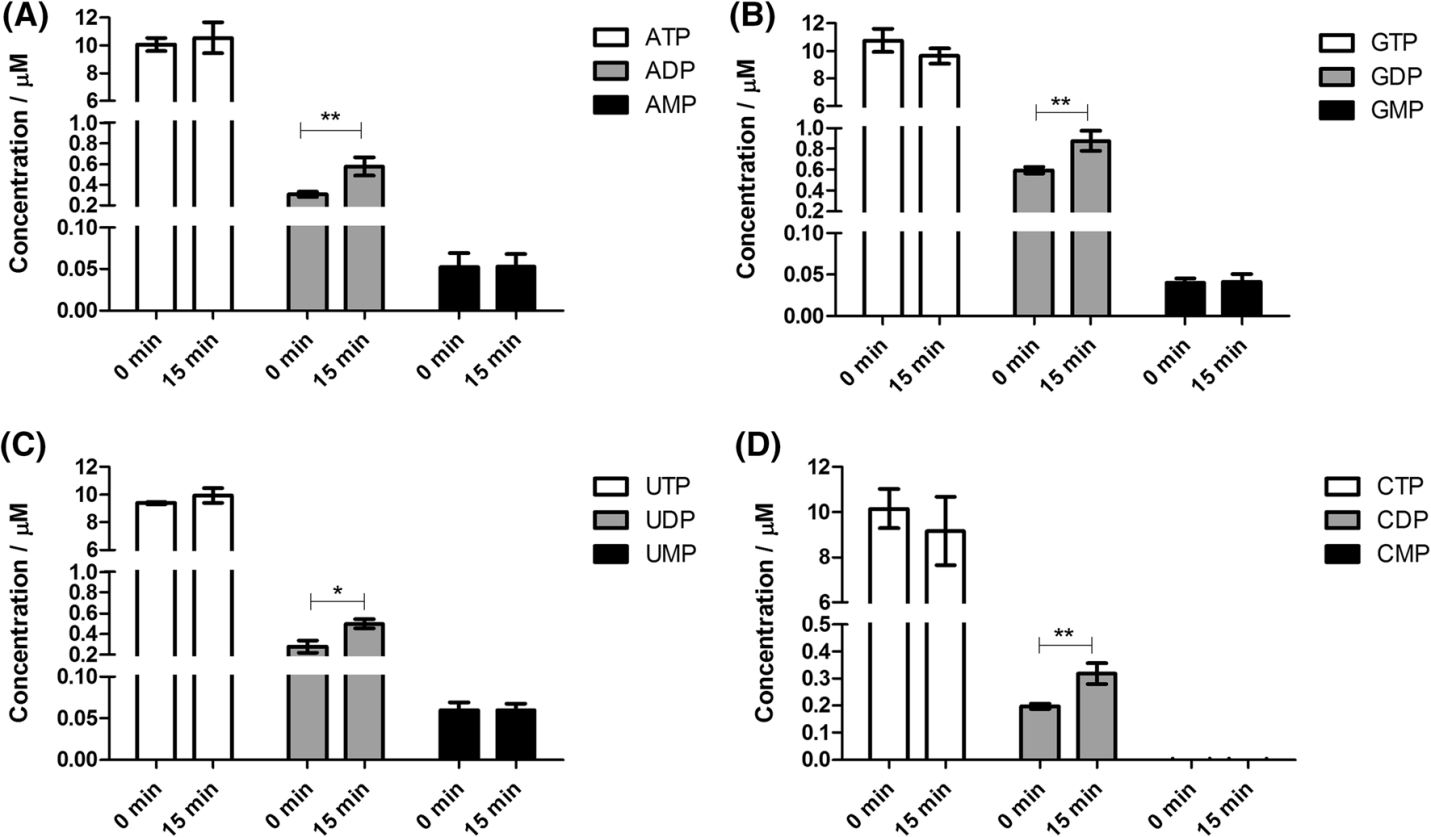
Effect of the culture medium on the degradation of nucleotide triphosphates. **a** ATP, **b** GTP, **c** UTP, and **d** CTP were incubated with Verduyn culture medium and extracted with 75% (aq.) boiling ethanol. Each bar represents the mean of four independently prepared samples (±SD). ANOVA: *p < 0.05; **p < 0.01 and ***p < 0.001

To discriminate between the stabilizing effect of the yeast matrix and that of the Verduyn culture medium on nucleotide triphosphates, we first evaluated the effect of the culture medium. The Verduyn culture medium is a mixture of organic compounds including glucose, a set of vitamins and a number of salts comprising divalent transition metal cations like Zn^2+^, Mn^2+^ and Cu^2+^, which have been shown to induce nucleotide degradation (Amsler and Sigel 1976; Sigel and Amsler 1976; Sigel and Hofstetter 1983; Verduyn et al. 1992). This effect may, however, be counteracted by the presence of chelating agents such as EDTA in the medium. Overall we found no stabilizing effect, since concentrations of nucleotide diphosphates increased significantly (*p* < 0.05; ANOVA) from 0.309 to 0.578 µM (87.1%) for ADP, 0.597 to 0.879 µM (47.2%) for GDP, 0.280 to 0.435 µM (55.3%) for UDP and 0.198 to 0.318 (60.6%) for CDP during boiling ethanol extraction (Fig. 4).

### Effect of yeast central carbon metabolites and some xenobiotic compounds on the stabilization/degradation of nucleotides

**Fig. 5:**
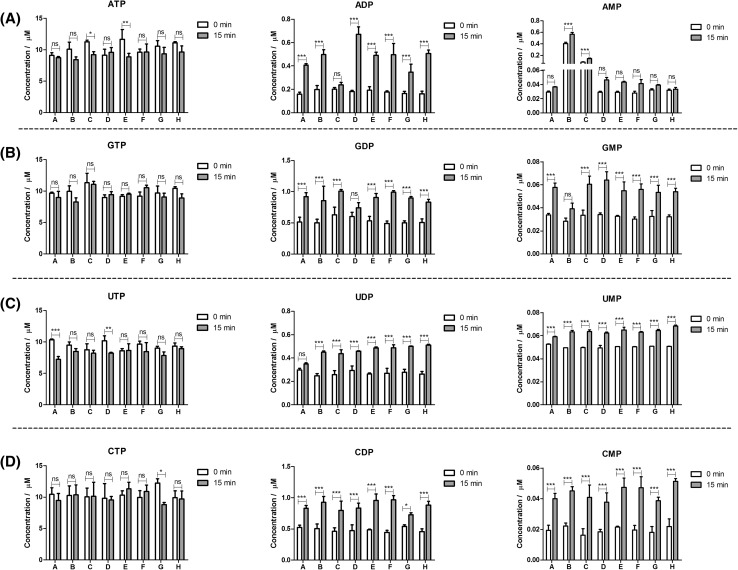
Effect of specific groups of central carbon metabolites on the degradation of nucleotide triphosphates. **a** ATP, **b** GTP, **c** UTP, and **d** CTP were incubated with 4 µL of solutions A–H (Table 1) and extracted with 75% (aq.) boiling ethanol. Each bar represents the mean of four independently prepared samples (±SD). ANOVA: *p < 0.05; **p < 0.01; ***p < 0.001 and *ns* not significant

We further investigated a range of central carbon metabolites as potential stabilizing factors including amino acids, organic acids, sugar phosphates, coenzymes, and other nucleotides that are typically found in the yeast matrix, as well as some yeast xenobiotic compounds (solutions A–H in Table 1). While the concentration of nucleotide triphosphates did not change significantly in the presence of these groups of compounds for the majority of cases (Fig. 5), solutions C and E for ATP, solutions A and D for UTP, and solution G for CTP showed a decrease in the concentration of the triphosphates after 15 min of extraction at 95 °C in boiling ethanol (Fig. 5). In comparison, concentrations of the diphosphates and most of the monophosphates increased indicating that these groups of compounds were not related to the stabilizing effect of the yeast matrix (*p* < 0.001; ANOVA; Fig. 5).

The increase in concentration of nucleotide diphosphates was differentially affected by the tested groups of compounds. The increase for ADP ranged from 18% in solution C to 272% in solution D, for GDP from 23% in solution D to 103% in solution F, for UDP from 17% in solution A to 95% in solution H, and for CDP from 35% in solution G to 119% in solution F. The increase in concentration of nucleotide monophosphates ranged from 4% in solution H to 81% in solution C for AMP, from 38% in solution B to 88% in solution D for GMP, from 12% in solution A to 34% in solution H for UMP, and from 103% in solution B to 150% in solution C for CMP (Fig. 5). These results contrast with the observation that the yeast matrix appears to stabilize the phosphoric acid anhydride bond against hydrolysis, as certain metabolites and components of solutions A–H actually promoted hydrolysis.

A number of observations indicated that we are not only dealing with the interconversion of triphosphates into di- and monophosphates. For example, there is a significant decrease in the concentration of ATP in solution E, which is not reflected in the much smaller increase in ADP (Fig. 5a). This indicates that while ATP is converted to ADP, there is a loss of ATP that cannot be accounted for. Another example is when ATP is incubated in solution D. While no reduction in ATP concentration was observed, there is a significant increase in ADP indicating that there is another source of ADP that is not derived from ATP. The increase in ADP (+272%) in solution D may be explained by the presence of NADP^+^, which according to Hofmann et al. (2010), can be thermally degraded into ADP. A similar thermal degradation mechanism may explain the increase of 155% in ADP in solution A, which contains NADPH (Fig. 5a). It is also noteworthy that the concentration of AMP, which did not change significantly (*p* > 0.05; ANOVA) in solutions A, D, E, F, G and H, was significantly elevated in solutions B and C (*p* < 0.001; ANOVA) even prior to the extraction process (t = 0 min; Fig. 5a). Degradation of NADH and NAD^+^ can produce AMP without ADP as an intermediate (Hofmann et al. 2010), which may explain the increase in AMP concentration prior to extraction. Furthermore, the presence of FAD might also contribute to this phenomenon.

These results show that the interconversion of energy metabolites is complex and cannot be solely addressed using stable isotope-dilution mass spectrometry. Gaining a better mechanistic understanding of the interconversion reactions under conditions of metabolite extraction is thus critical to obtain accurate results.

## Conclusions

We studied nucleotide triphosphates as representatives of energy metabolites under boiling ethanol conditions. The major findings were that degradation of nucleotide triphosphates into nucleotide di- and monophosphates is prevented by the yeast matrix as compared to standard solutions and that the interconversion of structurally-related metabolites such as NADPH and NADP^+^ into ADP and NADH, NAD^+^ or FAD into AMP may lead to erroneously elevated values that cannot be corrected for by the widely used stable-isotope-dilution mass spectrometry approach. To explain the matrix stabilization phenomenon, the influence of the culture medium (e.g. carbon source, vitamins, macro and micro elements) and particular groups of central carbon metabolites (amino acids, organic acids, sugar phosphates, coenzymes, and other nucleotides) was tested. While the basis of the observed stabilizing effect of the yeast matrix and possibly other cellular matrices remains unclear, we recommend studying the interconversion of metabolites in pilot experiments prior to large-scale metabolomics.

## Electronic supplementary material

Below is the link to the electronic supplementary material.

Supplementary material 1 (DOCX 434 kb)

Supplementary material 2 (TIFF 561 kb)

Supplementary material 3 (TIFF 200 kb)

Supplementary material 4 (TIFF 203 kb)

Supplementary material 5 (DOCX 19 kb)
