# Supplementary material for: The degradation of nucleotide triphosphates extracted under boiling ethanol conditions is prevented by the yeast cellular matrix
Source: Metabolomics. 2016 Nov 28;13(1):1. doi: 10.1007/s11306-016-1140-4 (PMC5126204; doi:10.1007/s11306-016-1140-4)
Supplement: Supplementary file 1 — Supplementary material 1 (DOCX 434 kb) [file 11306_2016_1140_MOESM1_ESM.docx]

**Electronic Supplementary Material**

**Materials and methods**

*Chemicals*

Acetonitrile (MeCN, HPLC SupraGradient grade) and ethanol (analytical reagent grade) were purchased from Biosolve (Valkenswaard, The Netherlands). All solvents and chemicals, as well as all standard compounds, including unlabeled and ^13^C^15^N-labeled ATP, GTP, CTP and UTP, were purchased from Sigma-Aldrich (Zwijndrecht, The Netherlands) at the highest available purity. ^13^C_6_-glucose was purchased from Cambridge Isotope Laboratories (Tewksbury, MA, USA). Ultrapure water was obtained from a Milli-Q Advantage A10 water purification system at a resistivity of 18.2 MΩ cm (Millipore Corp, Billerica, MA, USA).

*Calibration with ^13^C^15^N-labeled nucleotides*

For quantitative purposes ATP, ADP, AMP, GTP, GDP, GMP, CTP, CDP, CMP, UTP, UDP and UMP were weighed, dissolved in ultrapure water and mixed to obtain a single 12-analyte stock solution with a concentration of 100 µM per analyte. The stock solution was aliquoted and stored at −80°C until analysis. Standard calibration mixtures contained a fixed volume of 10% (^v^/_v_) of the ^13^C^15^N-labeled internal standard solution. Calibration curves were constructed based on the peak area ratios of unlabeled metabolites to their corresponding ^13^C^15^N-labeled standards between 9.8 nM and 10 µM.

*Statistical analysis*

To assess the repeatability of the experimental procedure, the extraction for each time point of the kinetics curves was performed four times (*n* = 4) in independently prepared samples. The statistical significance of differences between groups was evaluated by one-way ANOVA using GraphPad Prism version 5.0 for Windows (GraphPad Software, San Diego, CA, USA). The differences between the means were assessed using the Newman–Keuls multiple comparisons post-test and significance was determined at the 5% confidence level (*p <*0.05).

**Figures S-1 to S-3**

**
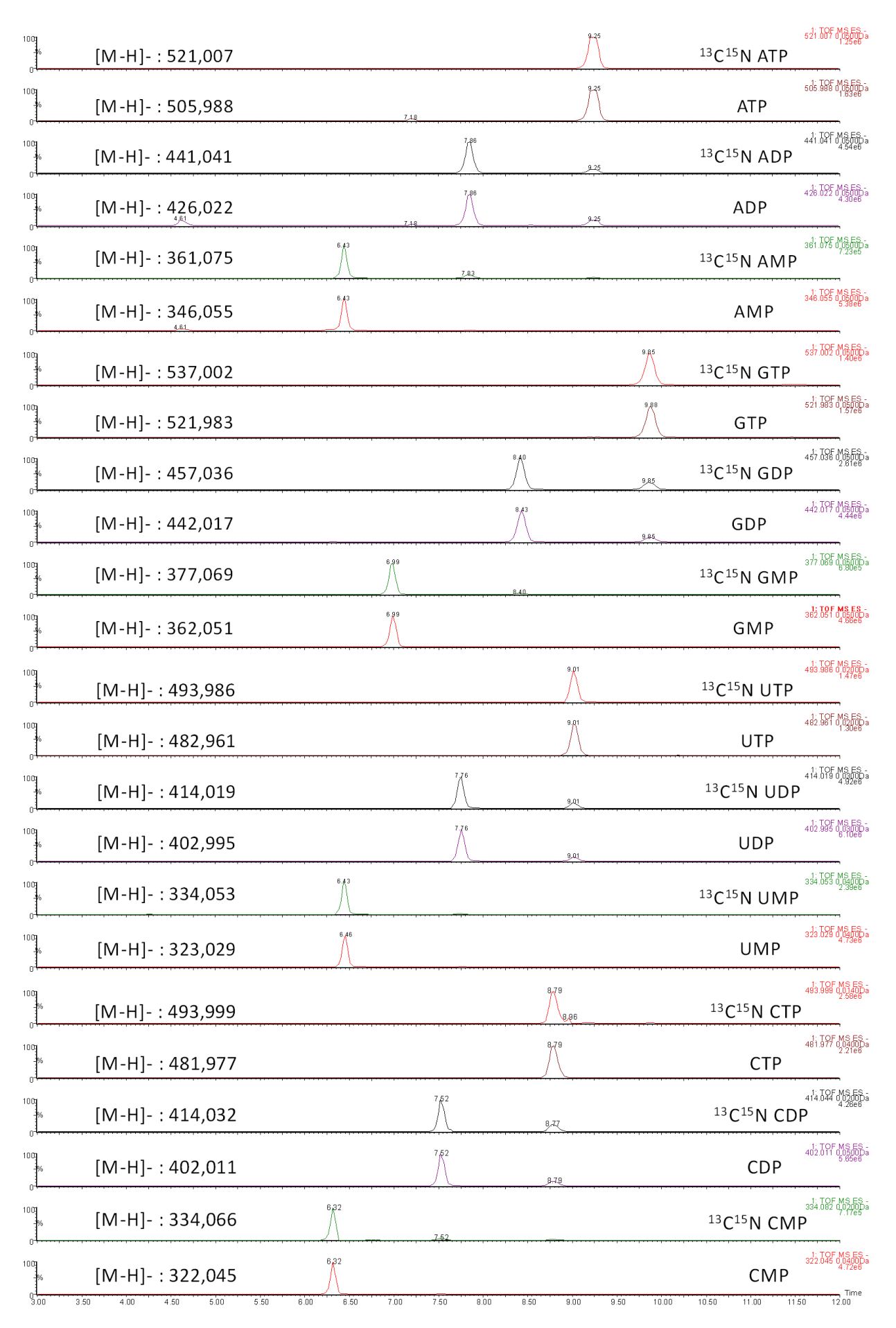
**

**Fig. S-1** Chromatographic separation of nucleotide tri-, di- and monophosphates and comparison of the smoothed EICs with the corresponding ^13^C^15^N-labeled standards.

**

**

**Fig. Fig. S-2** Calibration curves for purine nucleotide tri-, di- and monophosphates based on peak area ratios with the corresponding ^13^C^15^N-labeled internal standards. A) ATP, B) ADP, C) AMP, D) GTP, E) GDP and F) GMP.

**

**

**Fig. S-3** Calibration curves for pyrimidine nucleotide tri-, di- and monophosphates based on peak area ratios with the corresponding ^13^C^15^N-labeled internal standards. A) UTP, B) UDP, C) UMP, D) CTP, E) CDP and F) CMP.

**Table S-1** Validation parameters of the analytical method

| **Name** | **Calibration curve** | **r^2^** | **Repeatability^a^** | **Intermediate precision^a^** | **Accuracy^b^** |
| --- | --- | --- | --- | --- | --- |
| ATP | Y= 0.1270x + 0.0055 | 0.9982 | 3.28 | 11.8 | 95.5 (2.6) |
| ADP | Y= 0.0918x + 0.0035 | 0.9983 | 3.30 | 14.6 | 97.5 (3.1) |
| AMP | Y= 0.7477x + 0.0023 | 0.9980 | 3.51 | 13.8 | 96.5 (2.5) |
| GTP | Y= 0.1105x + 0.0014 | 0.9983 | 3.00 | 10.6 | 98.1 (3.4) |
| GDP | Y= 0.1676x - 0.0012 | 0.9984 | 3.27 | 10.4 | 95.4 (3.2) |
| GMP | Y= 0.6423x - 0.0067 | 0.9984 | 3.27 | 9.3 | 96.7 (3.2) |
| CTP | Y= 0.0854x -0.0001 | 0.9984 | 2.75 | 9.6 | 95.9 (3.9) |
| CDP | Y= 0.1352x - 0.0020 | 0.9983 | 3.26 | 13.6 | 97.2 (2.9) |
| CMP | Y= 0.6142x + 0.0029 | 0.9962 | 5.85 | 13.6 | 98.9 (3.3) |
| UTP | Y= 0.0923x - 0.0002 | 0.9979 | 3.31 | 6.6 | 100.1 (3.0) |
| UDP | Y= 0.1242x - 0.0026 | 0.9984 | 2.92 | 10.7 | 96.9 (3.8) |
| UMP | Y= 0.1976x - 0.0086 | 0.9977 | 3.65 | 7.0 | 99.7 (3.1) |

^a^ Data are presented as %CV

^b^ Data are presented as the average (n = 3) percent of accuracy, in parentheses %CV
