# Supplementary figures and images for: The degradation of nucleotide triphosphates extracted under boiling ethanol conditions is prevented by the yeast cellular matrix
Source: Metabolomics. 2016 Nov 28;13(1):1. doi: 10.1007/s11306-016-1140-4 (PMC5126204; doi:10.1007/s11306-016-1140-4)

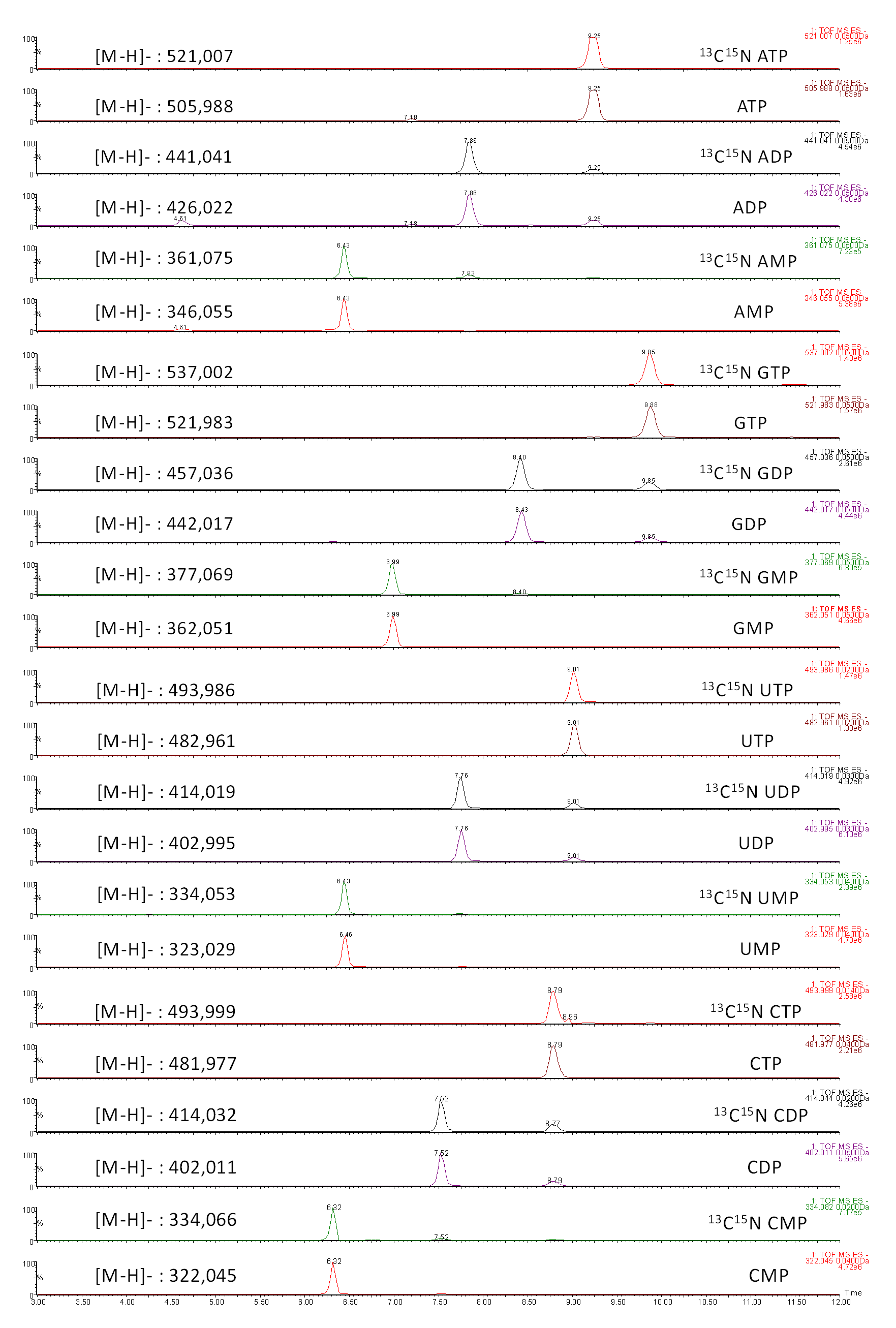

Supplement: Supplementary file 2 — Supplementary material 2 (TIFF 561 kb) [file 11306_2016_1140_MOESM2_ESM.tif]
