# Supplementary material for: The degradation of nucleotide triphosphates extracted under boiling ethanol conditions is prevented by the yeast cellular matrix
Source: Metabolomics. 2016 Nov 28;13(1):1. doi: 10.1007/s11306-016-1140-4 (PMC5126204; doi:10.1007/s11306-016-1140-4)
Supplement: Supplementary file 5 — Supplementary material 5 (DOCX 19 kb) [file 11306_2016_1140_MOESM5_ESM.docx]

**Table S-1** Validation parameters of the analytical method

| **Name** | **Calibration curve** | **r^2^** | **Repeatability^a^** | **Intermediate precision^a^** | **Accuracy^b^** |
| --- | --- | --- | --- | --- | --- |
| ATP | Y= 0.1270x + 0.0055 | 0.9982 | 3.28 | 11.8 | 95.5 (2.6) |
| ADP | Y= 0.0918x + 0.0035 | 0.9983 | 3.30 | 14.6 | 97.5 (3.1) |
| AMP | Y= 0.7477x + 0.0023 | 0.9980 | 3.51 | 13.8 | 96.5 (2.5) |
| GTP | Y= 0.1105x + 0.0014 | 0.9983 | 3.00 | 10.6 | 98.1 (3.4) |
| GDP | Y= 0.1676x - 0.0012 | 0.9984 | 3.27 | 10.4 | 95.4 (3.2) |
| GMP | Y= 0.6423x - 0.0067 | 0.9984 | 3.27 | 9.3 | 96.7 (3.2) |
| CTP | Y= 0.0854x -0.0001 | 0.9984 | 2.75 | 9.6 | 95.9 (3.9) |
| CDP | Y= 0.1352x - 0.0020 | 0.9983 | 3.26 | 13.6 | 97.2 (2.9) |
| CMP | Y= 0.6142x + 0.0029 | 0.9962 | 5.85 | 13.6 | 98.9 (3.3) |
| UTP | Y= 0.0923x - 0.0002 | 0.9979 | 3.31 | 6.6 | 100.1 (3.0) |
| UDP | Y= 0.1242x - 0.0026 | 0.9984 | 2.92 | 10.7 | 96.9 (3.8) |
| UMP | Y= 0.1976x - 0.0086 | 0.9977 | 3.65 | 7.0 | 99.7 (3.1) |

^a^ Data are presented as %CV

^b^ Data are presented as the average (n = 3) percent of accuracy, in parentheses %CV
